# How to Manage Suicidal Risk at Digital Distance

**DOI:** 10.1192/j.eurpsy.2022.183

**Published:** 2022-09-01

**Authors:** P. Courtet

**Affiliations:** University Hospital Montpellier, Emergency Psychiatry And Acute Care, Montpellier, France

**Keywords:** ecological momentary intervention, smartphone applications, Ecological Momentary Assessment

## Abstract

Suicide prevention remains very difficult to achieve for many reasons, notably because we do not have any indicator of risk prediction, short-term risk factors being little explored, and evaluations being retrospective they are biased. Furthermore, patients at risk are not followed up, because of their lack of confidence in care, stigma, shame. On the other hand, the gap observed during the covid19 pandemic between distress and less occurrence of suicides could be linked to more virtual contacts. Then, the smartphone might be a good tool to stay connected to a protective network. We will discuss the opportunity offered by the smartphone to monitor patients with ecological momentary assessment, allowing to better characterize their acute states and detect an increased risk in real time, and thanks to the ecological momentary intervention 24/7 availability, improve access to care and better coordinate resources, and encourage self-care. These tools while offering new solutions for an efficient real time suicide prevention, may also raise some ethical issues that should be addressed.

**Disclosure:**

Fondamental Foundation supported the developement of the application EMMA

